# Defective Mitochondrial Respiration in Hereditary Thoracic Aneurysms

**DOI:** 10.3390/cells14110768

**Published:** 2025-05-23

**Authors:** Daniel Marcos-Ríos, Antonio Rochano-Ortiz, Nerea Méndez-Barbero, Jorge Oller

**Affiliations:** 1 Laboratory of Vascular Pathology, Health Research Institute-Fundación Jiménez Díaz University Hospital, Universidad Autónoma de Madrid (IIS-FJD, UAM), 28040 Madrid, Spain; 2 Centro de Investigación Biomédica en Red de Enfermedades Cardiovasculares (CIBERCV), 28029 Madrid, Spain; 3 Facultad de Medicina, Universidad Alfonso X el Sabio (UAX), Villanueva de la Cañada, 28691 Madrid, Spain

**Keywords:** mitochondria, aneurysm, Marfan syndrome, Loeys-Dietz syndrome, familial thoracic aortic aneurysm, nicotinamide riboside, vascular smith muscle cells

## Abstract

Thoracic aortic aneurysms are life-threatening vascular conditions linked to inherited disorders such as Marfan syndrome, Loeys–Dietz syndrome, vascular Ehlers–Danlos syndrome, and familial thoracic aortic aneurysms and dissections. While traditionally associated with the extracellular matrix and contractile defects in vascular smooth muscle cells, emerging evidence suggests the key role of mitochondrial dysfunction. Here, we show that the overexpression of *ACTA2^R179H^* and *TGFBR2^G357W^* in murine aortic VSMCs reduces Mitochondrial Transcription Factor A (Tfam) expression, decreases mitochondrial DNA (mtDNA) content, and impairs oxidative phosphorylation, shifting metabolism toward glycolysis. Notably, nicotinamide riboside, a NAD^+^ precursor, restores mitochondrial respiration, increases Tfam and mtDNA levels, and promotes a contractile phenotype by enhancing actin polymerization and reducing matrix metalloproteinase activity. These findings identify mitochondrial dysfunction as a shared feature in hereditary thoracic aortic aneurysm, not only in Marfan syndrome, but also in other genetic forms, and highlight mitochondrial boosters as a potential therapeutic strategy.

## 1. Introduction

Different inherited disorders significantly affect the structural integrity of the aortic wall, resulting in thoracic aortic aneurysms (TAAs) [1]. This vascular condition is defined by the permanent dilation of the thoracic aorta, which, if not addressed, can lead to life-threatening complications such as aortic rupture or dissection, potentially resulting in fatal hemorrhaging [2]. Many of these disorders are associated with mutations in genes related to the extracellular matrix (ECM) or the contractile apparatus of vascular smooth muscle cells (VSMCs). Among these, Marfan syndrome (MFS) is one of the most prevalent connective tissue disorders, caused by mutations in the *FBN1* gene, which encodes the ECM protein, Fibrillin-1. Patients with MFS typically present elongated bones, *ectopia lentis*, and reduced life expectancy, largely due to the progression of TAAs [3]. Other genetic conditions impacting the aorta include Loeys–Dietz syndrome, which arises from mutations in genes involved in TGF-β signaling and shares certain skeletal characteristics with MFS. Vascular Ehlers–Danlos syndrome is mainly associated with mutations in the COL3A1 gene (which encodes collagen type III), leading to joint and skin hyperelasticity, short stature, and an increased risk of aortic dissection [4]. In familial nonsyndromic thoracic aortic aneurysms and dissections (FTAAD), while most causative genes remain unidentified, around 20% of cases are associated with mutations in genes that support the contractile apparatus of smooth muscle cells, including smooth muscle actin and myosin (ACTA2 and MYH11, respectively). The primary complication in all these inherited connective tissue disorders is the heightened risk of developing TAAs. Current management strategies for aortic aneurysms largely depend on surgical prophylactic repair [5]. Therefore, it is crucial to identify novel molecular mediators involved in the pathophysiology of TAAs to inform the development of new pharmacologic approaches.

VSMCs found in the medial layer of arteries, are essential for the contraction of the vascular wall and the regulation of vessel diameter. These cells can transition from a quiescent, contractile phenotype to a secretory phenotype, which is linked to increased proliferation, extracellular matrix (ECM) accumulation, and subsequent medial degeneration, all of which contribute to aneurysm formation [6]. Recent studies have begun to investigate the connections between cellular adhesion, cytoskeletal reorganization, and cellular metabolism, highlighting the critical role of ECM composition and stiffness in regulating metabolism. Our previous research has demonstrated that mitochondrial metabolism is a key regulator of the VSMC phenotype during aortic remodeling in Marfan syndrome and is finely tuned by ECM composition [7,8]. Furthermore, we have shown the significance of mitochondrial metabolism in aneurysm development in a conditional mouse model with specific mitochondrial dysfunction in VSMCs through the depletion of Tfam (mitochondrial transcription factor A) [8].

Based on these findings, we hypothesize that mitochondrial dysfunction in VSMCs is a key factor in aortic remodeling in Marfan syndrome and other types of hereditable-TAAs; therefore, restoring mitochondrial function could prevent aneurysm progression and aortic dissection. Additionally, enhancing mitochondrial function with nicotinamide riboside (NR), a precursor of NAD^+^, presents a promising therapeutic strategy for managing aortic aneurysms associated with Marfan syndrome, with the potential to prevent aortic dissection.

## 2. Material and Methods

### 2.1. Cell Procedures

The isolation and culture of primary vascular smooth muscle cells (VSMCs) from mice were carried out following the protocol described in [9]. Tissue digestion was performed using a solution containing collagenase (1 mg/mL) and elastase (0.5 mg/mL) from Worthington Biochemical until a single-cell suspension was achieved. All experiments using primary VSMCs were conducted between passages 2 and 4. Lentiviral transduction was performed over 5 h at a multiplicity of infection (MOI) of 3. Subsequently, the medium was replaced with fresh DMEM supplemented with 10% FBS, and the cells were cultured for an additional seven days. Cells were then treated with NR for three more days and serum-starved for 16 h. HEK-293T (CRL-1573) and Jurkat (Clone E6-1, TIB-152) cell lines were employed for the production of high-titer lentivirus and for titration, respectively. All cell lines were verified to be mycoplasma-free. For cell immunostaining, cells were fixed with 4% paraformaldehyde for 10 min and permeabilized with 0.3% Triton X-100 in PBS for 10 min. Samples were incubated with: 647-Phalloidin (1/500 dilution, Millipore, Burlington, NA, USA) and DAPI for 15 min. For immunofluorescence, the following antibodies were used: anti-Pgc1alpha (Novus Biologicals LLC, Zillow, Centennial, CO, USA), anti-Mt-Co2 (Proteintech, Rosemont, IL, USA), anti-Mt-Co1 (Invitrogene, Waltham, MA, USA), or anti-Tfam (Abcam, Cambridge, UK). Images were acquired using a Zeiss-LSM-800 microscope with a 40× oil immersion objective and the ZEN acquisition software v2.3.

### 2.2. Lentivirus Production and Infection

Lentiviruses expressing shRNA, which targets murine shFbn1, and control shRNA were purchased from Sigma-Aldrich (Burlington, MA, USA). Lentiviruses expressing ACTA2^R178H^ and TGFBR2^G317W^ mutations, along with control viruses, were generously provided by Mark Lindsay (Precision Cardiology Laboratory; Cardiovascular Research Center, Massachusetts General Hospital, Boston) [10]. Pseudotyped lentiviruses were generated via transient calcium phosphate transfection of HEK-293T cells and concentrated from the culture supernatant through ultracentrifugation (2 h at 128,000× *g*; Ultraclear Tubes; SW28 rotor and Optima L-100 XP Ultracentrifuge; Beckman, Brea, CA, USA). The viral particles were suspended in cold, sterile PBS and titrated by transducing Jurkat cells for 48 h. Transduction efficiency (measured as GFP-positive cells) and cell viability (using propidium iodide staining) were assessed by flow cytometry.

### 2.3. Extracellular Flux Analysis and Metabolic Assays

Oxygen consumption rates (OCRs) were measured using the XF-96 Extracellular Flux Analyzer (Seahorse Bioscience, North Billerica, MA, USA). A total of 25,000 mouse aortic VSMCs were seeded in unbuffered DMEM medium containing 25 mM glucose and 1 mM CaCl_2_. Baseline measurements were recorded three times, followed by sequential addition of oligomycin (1 mM), fluoro-carbonyl cyanide phenylhydrazone (FCCP; 1.5 mM), and a combination of rotenone (100 nM) and antimycin A (1 mM). Extracellular lactate levels were determined using the Accutrend^®^ Plus system (Roche, Basel, Switzerland), analyzing 20 μL of conditioned medium collected after 48 h of activation. Lactate concentrations were normalized to protein content in cell extracts.

### 2.4. Gelatin Zymography

Supernatants form cell cultures were prepared as described [9]. Extracts (15 μg) were fractioned under nonreducing conditions on 10% SDS–polyacrylamide gels containing 1% gelatin (Sigma). Gels were washed three times in 2.5% Triton X-100 for 2 h at room temperature; incubated overnight at 37 °C in 50 mM Tris-HCl pH 7.5, 10 mM CaCl_2_, and 200 mM NaCl; and stained with Coomasie Blue. The areas of gelatinolytic or MMP activity were visualized as transparent bands. Images were analyzed with Quantity One software v.2.6 (Bio-Rad, Hercules, CA, USA).

### 2.5. Quantitative PCR

Total RNA was extracted with TRIzol (Life Technologies, Carlsbad, CA, USA). To quantify mitochondrial DNA (mtDNA), total DNA from cells was isolated using the SurePrep kit (Fisher Scientific, Hampton, NH, USA) following the manufacturer’s protocols. DNA was amplified by real-time PCR using primers targeting cytochrome c oxidase subunit 1 (*mt-Co1*) and mitochondrial 16S rRNA and normalized to nuclear-encoded control genes, B2M and H2K. Quantitative PCR (qPCR) reactions were performed in triplicate using SYBR Master Mix (Promega, Madison, WI, USA), following the manufacturer’s instructions. Post-amplification melting curve analysis was conducted to verify probe specificity, ensuring a single melting temperature (Tm) peak per reaction. Relative mRNA quantification was carried out using the 2^−ΔCT^ method, with B2M, YWHAZ, and PP1A serving as normalization controls. Expression fold changes were calculated relative to control mRNA levels. qPCR was performed with the following primers:

*Tfam* CAGGAGGCAAAGGATGATTC; CCAAGACTTCATTTCATTGTCG

*Ppargc1a* GGCACGCAGCCCTATTCA; CGACACGGAGAGTTAAAGGAAGA

*mt-Co1* CTCGCCTAATTTATTCCACTTCA; GGGGCTAGGGGTAGGGTTAT

*Mmp2* CAAGTTCCCCGGCGATGTC; TTCTGGTCAAGGTCACCTGTC

*Mmp9* CACCACAGCCAACTATGACCA; CAGGAAGACGAAGGGGAAGAC

*Spp1* ATGAGATTGGCAGTGATTTG; CATCCTTTTCTTCAGAGGAC

*Col1a1* GCTCCTCTTAGGGGCCACT; CCACGTCTCACCATTGGGG

*16s Mt-rRNA* CCGCAAGGGAAAGATGAAAGAC; TCGTTTGGTTTCGGGGTTTC

*Hk2* nDNA GCCAGCCTCTCCTGATTTTAGTGT; GGGAACACAAAAGACCTCTTCTGG

*B2m* TACATACGCCTGCAGAGTTAAGCA; TGATCACATGTCTCGATCCCAG

*Pp1a* ACGCCACTGTCGCTTTTC; GCAAACAGCTCGAAGGAGAC

*Ywhaz* TTACTTGGCCGAGGTTGCT; TGCTGTGACTGGTCCACAAT

## 3. Results

**Figure 1:**
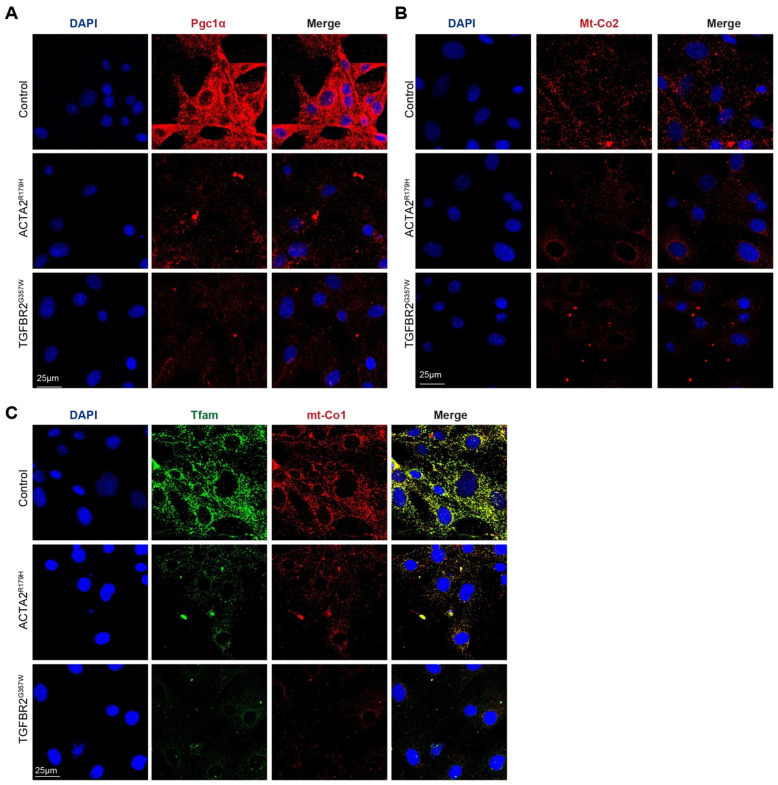
Mitochondrial proteins are downregulated in VSMC carrying thoracic aortic aneurysm mutations. Representative confocal images of (**A**) Pgc1a, (**B**) Mt-Co2, and (**C**) Tfam and Mt-Co1; in murnine VSMCs transduced with a control lentivector or with lentivectors overexpressing ACTA2R179 or TGFBR2G357W (to model FTAAD and LDS, respectively).

To investigate whether mitochondrial metabolism is affected in other thoracic aortic aneurysms and dissections (TAADs), we modeled familial thoracic aortic aneurysm and dissections (FTAAD) and Loeys–Dietz syndrome (LDS) by using lentivectors to overexpress human *ACTA2^R179H^* and *TGFBR2^G357W^* in primary murine aortic vascular smooth muscle cells (VSMCs). These constructs were generously provided by Mark Lindsay [10]. As shown in Figure 1, immunofluorescence analysis revealed a marked reduction in the protein levels of Pgc1α, Mt-Co2, Tfam, and Mt-Co1 in both *ACTA2^R179H^* and *TGFBR2^G357W^* mutant-transduced VSMCs compared to controls, suggesting impaired mitochondrial biogenesis and function in these disease models.

**Figure 2:**
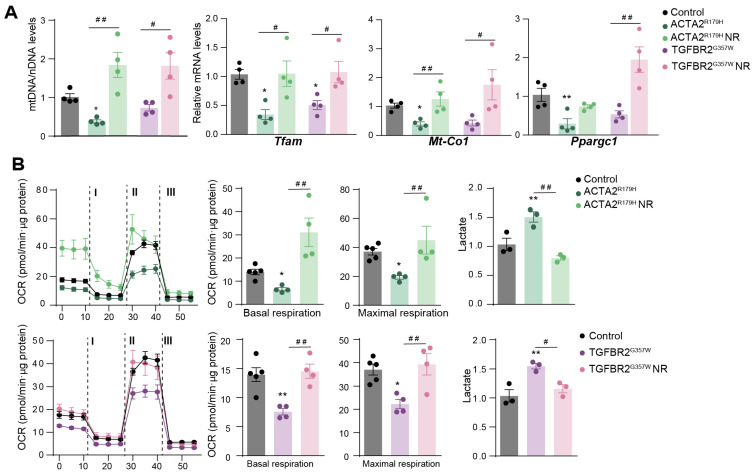
Nicotidamide riboside treatment improves mitochondrial function in VSMCs with thoracic aortic aneurysm mutations. The effect of NR on mouse primary VSMCs overexpressing ACTA2^R179^ or TGFBR2^G357W^ (to model FTAAD and LDS, respectively). VSMCs were insolated and transduced with lentivectors. After 2 days of transduction, VSMCs were treated with NR (0.25 mg/mL) for 5 days. (**A**) qPCR analysis of relative mtDNA content and RT–qPCR analysis of *Tfam*, *Mt-Co1* and *Ppargc1a* mRNA expression. (**B**) OCR measured using Seahorse^Tm^ at basal respiration and after addition of oligomycin (I) and FCCP (II) to measure maximal respiration, followed by a combination of rotenone and antimycin A (III). The right-most panel shows extracellular lactate levels. Data are mean ± s.e.m. Statistical significance was assessed by one-way ANOVA: * *p* < 0.05, ** *p* < 0.01 vs. Control; # *p* < 0.05, ## *p* < 0.01 vs. NR treatment.

The overexpression of *ACTA2^R179H^* and *TGFBR2^G357W^* resulted in reduced levels of Tfam mtDNA and decreased the mRNA expression of mitochondrial regulators (Figure 2A). Flux analysis of the oxygen consumption rate (OCR), an indicator of mitochondrial oxidative phosphorylation, demonstrated diminished mitochondrial respiration in TAA-VSMCs (Figure 2B). This decline in mitochondrial function was accompanied by increased extracellular lactate production, suggesting a shift toward glycolysis as the primary source of cellular energy in the presence of TAAD mutations (Figure 2B). Notably, incubation with nicotinamide riboside (NR) reversed the impact of both mutations on these metabolic parameters (Figure 2A,B).

**Figure 3:**
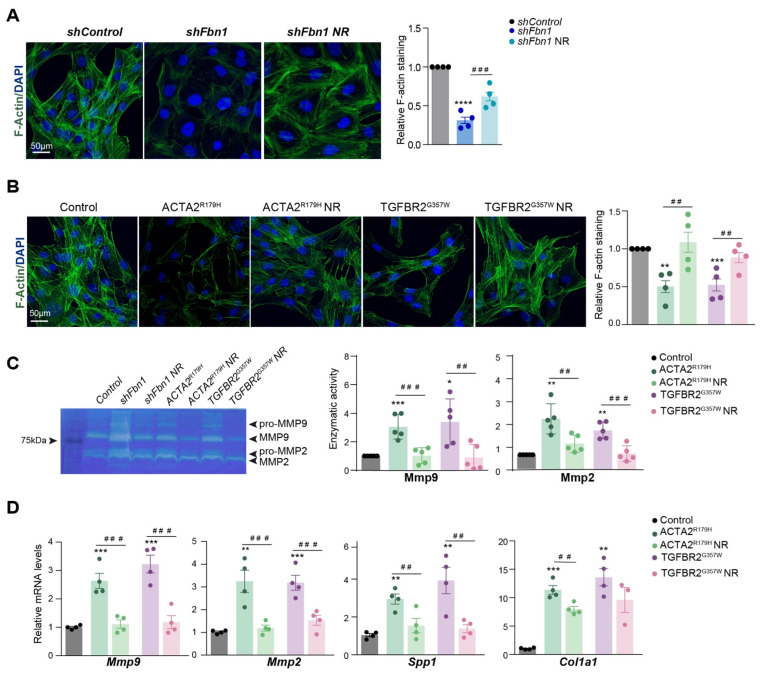
Nicotinamide riboside treatment restores the balance between the synthetic and contractile phenotypes in VSMCs with thoracic aortic aneurysm mutations. Effect of NR on mouse primary VSMCs silenced for Fbn1 (*ShFbn1*), overexpressing ACTA2^R179^ or TGFBR2^G357W^ (to model MFS, FTAAD, and LDS, respectively). VSMCs were isolated and transduced with lentivectors. After 2 days of transduction, VSMCs were treated with NR (0.25 mg/mL) for 5 days. (**A**) Representative confocal imaging of filamentous actin (F-actin) stained with phalloidin (green); nuclei are stained with Dapi (Blue); (n = 4) and quantification in murine VSMCs silenced for *Fbn1* (*ShFbn1* or control shRNA, *shControl*). (**B**) Representative confocal imaging of filamentous actin (F-actin) stained with phalloidin (green); nuclei are stained with Dapi (Blue); (n = 4) quantification in murine VSMCs in VSMCs overexpressing ACTA2^R179^ or TGFBR2^G357W^. (**C**) Representative Gelatin-zymography analysis of Mmp2 and Mmp9 activity in 24 h in conditional medium and quantification. (**D**) RT-qPCR analysis of *Mmp9*, *Mmp2*, *Spp1*, and *Col1a1* mRNA expression. Data are mean ± s.e.m. Statistical significance was assessed by one-way ANOVA: * *p* < 0.05, ** *p* < 0.01, *** *p* < 0.001, **** *p* < 0.0001 vs. Control, ## *p* < 0.01, ### *p* < 0.001, vs. NR treatment.

Furthermore, NR enhanced VSMC-contractile phenotypes, as indicated by increased F-actin polymerization in FBN1-silenced cells modeling Marfan syndrome, as well as in *ACTA2^R179H^* and *TGFBR2^G357W^* VSMCs (Figure 3A,B), while also reducing the expression and activity of matrix metalloproteinases and other secretory markers (Figure 3C,D).

## 4. Discussion

Our findings suggest that reduced Tfam expression and mtDNA levels induce a metabolic shift from oxidative phosphorylation (OXPHOS) to glycolysis in VSMCs affected by familial TAAs. This metabolic reprogramming is increasingly recognized as a hallmark of aneurysm pathogenesis, contributing to vascular remodeling and disease progression [8,11,12]. Mitochondrial dysfunction alters bioenergetics and cellular processes, such as apoptosis and matrix remodeling, exacerbating aneurysm development [8].

Mutations such as ACTA2^R179^ and TGFBR2^G357W^ are thought to contribute to mitochondrial dysfunction through distinct but interconnected mechanisms.

The ACTA2^R179^ mutation affects smooth muscle α-actin, a critical component of the cytoskeleton, potentially disrupting cytoskeletal organization and impairing mitochondrial dynamics, such as fission, fusion, and transport. Altered cytoskeletal integrity can compromise mitochondrial positioning and energy distribution within cells, leading to reduced mitochondrial function [13,14,15].

Meanwhile, TGFBR2^G357W^ affects a key receptor in the TGF-β signaling pathway, which regulates extracellular matrix production, cell survival, and oxidative stress responses. Dysregulation of TGF-β signaling can impair mitochondrial biogenesis, by increasing reactive oxygen species (ROS) production, promoting mitochondrial DNA damage, all contributing to mitochondrial dysfunction [16,17,18]. Moreover, a TGF-β-activated response has been reported during ECM remodeling, leading to mitochondrial fission and mitochondrial unfolded protein response (UPRMT) [19].

Improving mitochondrial fitness in vitro restored the contractile phenotype of these cells, supporting the idea that mitochondrial dysfunction plays a causative role in TAA pathogenesis. This aligns with prior studies showing that mitochondrial-targeted therapies can improve vascular smooth muscle function and reduce maladaptive phenotypic switching [8,20,21,22].

Notably, nicotinamide riboside (NR) increased Tfam expression, mtDNA levels, and mitochondrial metabolism while reducing the secretory phenotype in TAA VSMCs. These findings suggest that NR may counteract the glycolytic shift observed in TAA VSMCs, potentially reducing disease progression.

Several strategies to boost NAD⁺ levels included direct supplementation with NAD⁺ precursors, stimulation of key enzymes involved in NAD⁺ biosynthesis, and prevention of intermediate loss within the NAD⁺ production pathway [23]. NR, a precursor of NAD+, is known to promote mitochondrial biogenesis and improve metabolic function therapeutic potential of mitochondrial-targeted agents. NR has been explored in other cardiovascular disorders, including genetic hypertrophic cardiomyopathy, where enhanced mitochondrial NAD⁺ levels in patient myectomy samples have shown improvements in mitochondrial performance [24].

Our results reinforce the idea that metabolic remodeling is a key driver of hereditary TAA progression. Given the limited therapeutic options, targeting mitochondrial metabolism, particularly with NAD^+^ boosters, could provide a promising strategy for treating genetic aortopathies [23]. However, further studies are needed to explore the long-term efficacy of mitochondrial-targeted therapies in aneurysm prevention and treatment.

## 5. Conclusions

Our findings reveal that mitochondrial dysfunction is a common pathological mechanism in hereditary thoracic aortic aneurysm, encompassing Marfan syndrome and other genetic variants, thereby positioning therapies aimed to boost mitochondrial function as a promising therapeutic avenue.

## Data Availability

The data materials and methods supporting the findings of this study are available from the corresponding author on request.
